# Determination of biological and physicochemical parameters of *Artemia franciscana *strains in hypersaline environments for aquaculture in the Colombian Caribbean

**DOI:** 10.1186/1746-1448-1-9

**Published:** 2005-10-26

**Authors:** William N Camargo, Gabriel C Durán, Orlando C Rada, Licet C Hernández, Juan-Carlos G Linero, Igor M Muelle, Patrick Sorgeloos

**Affiliations:** 1 Fisheries and Illinois Aquaculture Center, Southern Illinois University Carbondale, Carbondale, IL 62901, USA; 2 Escuela Superior Politécnica del Litoral (ESPOL), Guayaquil, Ecuador; 3 Grupo de Investigación de la Artemia (GIA), Universidad del Atlántico y Fundación Universitaria San Martín, Barranquilla, Colombia; 4 Departamento de Biología, Universidad del Atlántico, Barranquilla, Colombia; 5 Artemia Reference Center and Laboratory of Aquaculture, University of Ghent, Rozier 44, Ghent B-9000, Belgium

## Abstract

**Background:**

*Artemia *(Crustacea, Anostraca), also known as brine shrimp, are typical inhabitants of extreme environments. These hypersaline environments vary considerably in their physicochemical composition, and even their climatic conditions and elevation. Several thalassohaline (marine) environments along the Colombian Caribbean coast were surveyed in order to contribute to the knowledge of brine shrimp biotopes in South America by determining some vital biological and physicochemical parameters for *Artemia *survival. Additionally, cyst quality tests, biometrical and essential fatty acids analysis were performed to evaluate the economic viability of some of these strains for the aquaculture industry.

**Results:**

In addition to the three locations (Galerazamba, Manaure, and Pozos Colorados) reported in the literature three decades ago in the Colombian Caribbean, six new locations were registered (Salina Cero, Kangaru, Tayrona, Bahía Hondita, Warrego and Pusheo). All habitats sampled showed that chloride was the prevailing anion, as expected, because of their thalassohaline origin. There were significant differences in cyst diameter grouping strains in the following manner according to this parameter: 1) San Francisco Bay (SFB-Control, USA), 2) Galerazamba and Tayrona, 3) Kangarú, 4) Manaure, and 5) Salina Cero and Pozos Colorados. Chorion thickness values were smaller in Tayrona, followed by Salina Cero, Galerazamba, Manaure, SFB, Kangarú and Pozos Colorados. There were significant differences in naupliar size, grouping strains as follows (smallest to largest): 1) Galerazamba, 2) Manaure, 3) SFB, Kangarú, and Salina Cero, 4) Pozos Colorados, and 5) Tayrona. Overall, cyst quality analysis conducted on samples from Manaure, Galerazamba, and Salina Cero revealed that all sites exhibited a relatively high number of cysts.g^-1^. Essential fatty acids (EFA) analysis performed on nauplii from cyst samples from Manaure, Galerazamba, Salina Cero and Tayrona revealed that cysts from all sites exhibited high arachidonic acid:20:4(*n*-6) (ArA) and eicosapentaenoic acid: 20:5(*n*-3) (EPA) levels comparable to the control sample (SFB). In contrast, most cysts collected (including SFB) at different locations, and during different months, presented low docosahexaenoic acid: 22:6(*n*-3) (DHA) levels (Manaure was the only exception with high DHA levels). Some variations in EPA and ArA levels were observed in all sites, contrasting with the much lower DHA levels which remained constant for all locations, except for Manaure which exhibited variable DHA levels. DHA/EPA ratio was overall very low for all sites compared to SFB cysts. All strains had a low DHA/ArA, but a high EPA/ArA ratio, including the control.

**Conclusion:**

The Colombian *A. franciscana *habitats analyzed were determined to be thalassohaline, and suitable for *A. franciscana *development. EFA profiles demonstrated that Tayrona, Galerazamba, Manaure and Salina Cero strains are suitable food for marine fish and crustacean culture because of their high EPA/ArA ratio, but might have to be fortified with DHA rich emulsions depending on the nutritional requirements of the species to be cultured, because of their overall low DHA content. The relatively small nauplii are appropriate for marine larvaeculture. In contrast, the strains from Tayrona, Kangarú, Salina Cero, and Pozos Colorados may be of use but limited to *Artemia *small biomass production quantities, because of the small surface area of their respective locations; *Artemia *could be exploited at these locations for local aquaculture applications. In general, cyst quality evaluation for Manaure, Salina Cero and Galerazamba cysts revealed that cysts from these three locations could improve their quality by concentrating efforts on cyst processing techniques. Finally, most locations had great *A. franciscana *production potential and require different degrees of water quality and/or infrastructure management.

## Background

Members of the genus *Artemia *(Crustacea, Anostraca), also known as brine shrimp, are typical inhabitants of extreme environments that have low species diversity and simple trophic structures [1]. These hypersaline environments vary considerably in terms of ionic composition, climatic conditions and altitude. As a general rule, chloride-rich lakes are the most adequate for *Artemia *development [2]; however, some strains require carbonate- (Mono lake *Artemia*, USA) or sulfate-rich waters (Tso Kar Lake *Artemia*, Tibet) for survival [1,3-5]. Conversely, some other ions may be deleterious to *Artemia*; potassium could be very toxic because of its occurrence with sodium [6,7]. Since water composition is important for *Artemia *survival, ecosystems where it occurs were classified in three categories based on their anionic composition: chloride-, sulfate- and carbonate-rich [2].

*Artemia persimilis *(Piccinelli and Prosdocimi 1968) and *A. franciscana *(Kellogg 1906) occur in waters of the American continent; the latter is the most cosmopolitan [8,9]. However, *A. persimilis *was reported in Sardinia, Italy [10]. Molecular (RAPD-randomly amplified polymorphic DNA) [11] and morphometric characters [12] indicate that *A. franciscana *occurs in the Colombian Caribbean.

To characterize *Artemia *strains the aquaculture industry employ an array of evaluation tools [13,14]: *i) Cyst and nauplii biometry*: cyst biometry assists in the determination of number of cysts.g^-1^; generally, 1 g from strains that produce small cysts contains more cysts.g^-1^, thus usually produce more nauplii.g^-1^. Similarly, naupliar biometry is also an essential tool for quality evaluation. Shorter naupliar length is important, particularly, to feed fish larvae which contrary to crustaceans, has to engulf prey in a single bite; *ii) Cyst hatching characteristics*: it can be affected by environmental factors, genotypical conditions and/or improper processing/storage. An acceptable cyst product should contain minimal quantities of impurities (i.e. sand, salt crystals, etc.) [13]. Hatching efficiency (HE) and hatching percentage (H%) vary greatly among commercial batches and account for most of the price difference [13]. However, HE may be a better criterion than H% since HE considers impurities content (i.e. empty cyst shells). Hatching values for a commercial product may be as low as 100,000 nauplii.g^-1^, but might yield ideally near 300,000 nauplii.g^-1 ^(with H% > 90). Hatching synchrony must be high (T_s _= 12–16 h), and the last nauplii should hatch within 8 h after T_90 _[13]. When T_s _is low (T_0_–T_100 _> 10 h), first hatched nauplii will have consumed most of their energy reserves by the time that the last nauplii hatched and harvesting has ended [13]; and *iii) Essential fatty acid (EFA) profiles*: particular attention has been given by marine larvaeculture production facilities to search for EFA rich *Artemia *strains, i.e. arachidonic acid:20:4(*n*-6) (ArA), eicosapentaenoic acid: 20:5(*n*-3) (EPA), and docosahexaenoic acid: 22:6(*n*-3) (DHA) [15]. Since ArA and DHA are vital in marine fish nutrition [16-18] great effort has been devoted to incorporate high levels of ArA, DHA, and high ratios of DHA/EPA/ArA in live food. ArA is generally conserved during periods of starvation in marine fishes [19], and serves as the preferred precursor for eicosanoid biosynthesis [20]. *Artemia *fed with enriched *n*-3 and *n*-6 highly unsaturated fatty acids (HUFA) in turn results in better larval growth and survival of several marine species fed with it [18,21-25]. The DHA/EPA ratio is very variable in non-enriched *Artemia*, with values often lower than 1. Through the addition of DHA rich emulsions the DHA/EPA ratio increases up to 7 [15]. Enrichment success is strain dependent (i.e. particular Chinese strains), and linked to variations in DHA catabolism. Further, EFA nutritional requirements may vary between species and developmental stages [18]. In white bass larvae, the optimal DHA/ArA and EPA/ArA dietary ratios have been established at 2:1 and 1:1, respectively [26]. This contrasts with flat fish larvae (i.e. turbot and Atlantic halibut) which require much higher ratios of over 10:1 [27,28]. However, high ArA levels have been implicated in the malpigmentation of various flatfish species [29]. An additional consideration is the possibility of DHA deficiency in neural tissues (i.e. vision) in larvae fed fish oil-based diet, as has been observed in Atlantic halibut larvae and juvenile herring eyes [30,31].

Our objective was to characterize *Artemia franciscana *biotopes in Colombia and to evaluate the viability of some strains for commercial exploitation. The data presented here may be of importance for the aquaculture industry to find new *Artemia *sources.

## Results

Along the Colombian Caribbean coast, nine potential *Artemia *habitats were explored with variable quantities (from very few cysts in the sediment to several pounds dispersed along the pond edges) of cysts and/or biomass and variable surface area (2.5 Tayrona to 4000 ha Manaure).

There were significant differences (Table 1) in cyst diameter (P = 0.00001), grouping strains in the following manner according to this parameter: 1) San Francisco Bay (SFB-Control ARC1258, USA), 2) Galerazamba and Tayrona, 3) Kangarú, 4) Manaure, and 5) Salina Cero and Pozos Colorados. Chorion thickness from Tayrona was the thinnest, followed by Salina Cero, Galerazamba, Manaure, SFB, Kangarú, and Pozos Colorados.

**Table 1:** Biometric determination of *Artemia franciscana *cysts and Instar I nauplii samples from several strains in the Colombian Caribbean and from San Francisco Bay (control ARC 1258) (units in μm).

Location	Cysts diameter	Chorion thickness	Nauplii length
SFB (ARC 1258)	201.0^a ^± 15.8 / 183.4 ± 6.9	8.8	432.1^a ^± 24.6
Galerazamba	232.1^b ^± 16.5 / 214.8 ± 19.3	8.6	390.3^b ^± 24.5
Tayrona	233.4^b ^± 12.4 / 227.2 ± 8.2	3.1	451.9^c ^± 25.1
Kangarú	236.8^c ^± 12.3 / 212.0 ± 11.0	12.4	426.1^a ^± 26.4
Manaure	241.1^d ^± 12.1 / 223.9 ± 10.2	8.6	414.2^d ^± 29.3
Salina Cero	249.8^e ^± 10.5 / 234.2 ± 10.1	7.8	431.7^a ^± 31.4
Pozos Colorados	252.9^e ^± 10.7 / 226.5 ± 10.2	13.2	442.0^e ^± 24.0

Superscripts (a, b, c, d, e) per column denote significant differences among strains (P < 0.05).

Note: Cyst diameter represents both non-decapsulated and decapsulated values, respectively.

There were significant differences (Table 1) in naupliar size (P = 0.0001) where strains grouped as follows (smallest to largest): 1) Galerazamba, 2) Manaure, 3) SFB, Kangarú, and Salina Cero, 4) Pozos Colorados, and 5) Tayrona.

Overall, *Manaure*: had a high number of cysts.g^-1 ^(Table 2) and HE, but a low H% and T_s_. *Galerazamba: *had a low number of cysts.g^-1 ^compared to other commercial cyst types, and a low H%, contrasting with a high HE and T_s_. *Salina Cero: *had a high number of cysts.g^-1^, but a low H%, HE and T_s_. SFB (control): had a high number of cysts.g^-1^, HE and H%, but a low T_s_.

**Table 2:** Quality evaluation results for *Artemia franciscana *cyst from three major saltworks in the Colombian Caribbean (H%: hatching percentage, HE: hatching efficiency, HR: hatching rate, Ts: hatching synchrony).

Location	Number of cysts/g*	**H% **(nauplii from 100 full cysts)	**HE **(nauplii/g of cyst)	**HR **(hrs)			
				
				**T**_**0**_	**T**_**10**_	**T**_**90**_	**T**_**s**_
Manaure	267,970.3 ± 5,639^a^	51.4 ± 0.6^a^	155,555.6 ± 6.3^a^	12	13	23.0	10.0
Galerazamba	208,260.4 ± 7,485^b^	53.1 ± 8.3^a^	125,888.9 ± 10.9^b^	12	13	26.0	13.0
Salina Cero	230,680.3 ± 4,474^c^	46.7 ± 2.1^b^	98,666.7 ± 2.2^c^	12	13	23.0	10.0
SFB (ARC1258)	283,556.1 ± 3,967^d^	67.4 ± 14.9^c^	127,222.2 ± 22.8^d^	15	16	25.5	9.5

* Mean values.

Superscripts (a, b, c, d) per column denote significant differences among strains (P < 0.05).

From the three (Cl^-^, SO_4_^2- ^and CO_3 _^2-^) characteristic anions used to classify hypersaline ecosystems [2], Cl^- ^was the most abundant anion (Table 3) in all locations evaluated. The physicochemical parameters monitored (Table 4) presented some tendencies inherent to each site. Salinity in Pozos Colorados and Salina Cero had a tendency to maintain low salinities (rarely crystallizing), contrasting with Manaure that presented salinities close to crystallization in the evaporation portion of the salt production circuit. Similarly, pH in Galerazamba, Salina Cero and Manaure was towards the low end pH for *Artemia *production; while for Tayrona and Pozos Colorados it was towards the ideal pH (8.0 to 8.5). Percent O_2 _saturation was overall normal in most sites, with the exception of Tayrona which was rather low in some months. Water temperature was at the upper limit in most sites, and extremely high only in Pozos Colorados. Nitrite was overall low in all sites, contrasting with high nitrate concentration in all sites. Phosphate was also low, except in Pozos Colorados where it was too high. Primary production (chlorophyll *a*) was rather at the low end for hypersaline ecosystems. Precipitation was high in the southern sites explored (as expected) and low at northernmost locations (dessert-like sites).

**Table 3:** Characteristic anion composition of all extreme environments where *Artemia franciscana *has been reported in the Colombian Caribbean. (Gz: Galerazamba saltwork, SC: Salina Cero lagoon, Kan: Kangarú salt pond, PC: Pozos Colorados saltwork, Tay: Chengue salt pond in the Tayrona Natural National Park, Ma: Manaure saltwork, BH: Bahía Hondita saltern, Pu: Pusheo saltern, Warrego was dried, thus not in table) (units in g/l).

Anions	Gz	SC	Kan	PC	Tay	Ma	BH	Pu
Cl^-^	55.00	11.86	8.00	60.00	75.00	137.50	11.50	35.00
SO_4_^2-^	12.90	3.36	*	3.47	3.78	11.14	8.24	3.98
HCO_3_^-^	0.11	0.19	0.14	0.29	0.97	0.23	0.11	0.21
CO_3_^2-^	*	*	0.176	*	*	*	*	*

* Below detection limit.

Note: Ionic concentrations represent one single sample per pond or salt concentration basin at the sites where *Artemia *was reported and must not be used for comparison purposes (between locations), since ionic concentration might vary periodically especially in managed (saltworks with several concentration levels as well as different viscosities in the system) compared to unmanaged ecosystems (single evaporation basin).

**Table 4:** Physicochemical parameters of seven locations in the Colombian Caribbean where *Artemia franciscana *strains inhabit. Salinity range, pH range, temperature range, nutrients range (NO_2_^-^, NO_3_^- ^and PO_4_^-3^), max. precipitation (month), and Chl *a *(sites sampled monthly between July 1998 and June 2000) (n = 20 stations per location).

*Parameters*	*Galerazamba*	*Salina Cero*	*Pozos Colorados*	*Tayrona*	*Manaure*	*Pusheo*	*Bahía Hondita*
Salinity (g/l)	65 – 295	19 – 204	5 – 291	34 – 330	148 – 275	40	15
pH	7.2 – 8.1	6.7 – 8.6	7.4 – 8.9	7.9 – 8.8	7.6 – 7.9	8.5	8.4
Percent O_2 _sat.	70 – 150	53 – 131	66 – 212	23 – 131	56 – 99	ND	ND
Temp. (C)	26.6 – 35.5	27.5 – 35.1	26.7 – 38.5	23.4 – 33.8	24.9 – 31.3	27.2	26.5
NO_2_^-^(mg/l)	0.005 – 0.120	0.003 – 0.115	0.001 – 0.077	0.002 – 0.018	0.005 – 0.025	0.007	0.073
NO_3_^-^(mg/l)	1.4 – 33.7	0.4 – 18.8	1.7 – 19.5	2.3 – 22.1	0.3 – 20.5	4.7	13.6
PO_4_^-3 ^(mg/l)	0.33 – 1.98	0.21 – 5.05	0.01 – 18.5	0.32 – 2.83	0.05 – 1.27	1.03	2.52
Chl. *a *(mg/m^3^)	0.01 – 0.11	0.09 – 3.04	0.002 – 2.72	0.01 – 0.39	0.09 – 0.10	ND	ND
Max. Precipitation (mm/month)	326.7	326.7	288.2	288.2	79.6	79.6	79.6
Total months sampled	24	22	13	18	24	1	1

Cyst samples from locations (Table 5) where enough cysts were collected to perform FAME analysis (FAME was actually done on freshly hatched nauplii from cysts), exhibited high EPA and ArA levels comparable to control sample (SFB-ARC1258). In contrast, most cysts collected (including SFB) at different locations, and during different months, presented low DHA levels (Manaure was the only exception with high DHA levels). Some variations in EPA and ArA levels were observed in all sites, contrasting with much lower DHA levels which remained constant for all locations, except for Manaure which exhibited variable DHA levels. DHA/EPA ratio was overall very low for all sites compared to SFB cysts. All strains had a low DHA/ArA ratio, but a high EPA/ArA ratio, SFB included.

**Table 5:** Intra-strain variability of ArA 20:4(*n*-6), EPA 20:5(*n*-3), and DHA 22:6(*n*-3) of some freshly hatched *Artemia franciscana *nauplii. Cysts samples collected in the Colombian Caribbean from 1998 to 2000 (Tay: Tayrona, Gz: Galerazamba, Ma: Manaure, SC: Salina Cero, SFB: San Francisco Bay control ARC1258) (values expressed in area %).

EFA	Tay	Gz	Ma	SC	SFB
**EPA **20:5(*n*-3)	2.7 – 3.6	0.3 – 8.6	1.7 – 3.1	2.2 – 5.9	0.3–2.4
**DHA **22:6(*n*-3)	0.1	0.1 – 0.3	0.1 – 1.3	0.1	0.4
**ArA **20:4(*n*-6)	2.8–3.4	0.1–3.9	0.9–1.1	1.3–3.2	0.9–1.3
**DHA/EPA**	0.03–0.04	0.03–0.33	0.06–0.42	0.02–0.05	0.03–1.33
**EPA/ArA**	0.96–1.06	2.21–3.00	1.89–2.82	1.69–1.84	0.33–10.23
**DHA/ArA**	0.03–0.04	0.08–1.00	0.11–1.18	0.03–0.08	0.31–0.44
Months sampled*	(2)	(9)	(3)	(4)	(2)

*Each sample was taken from different months.

Note: Values express max.-min. Ranges expressed as percentages of total EFA.

## Discussion

Vanhaecke and Sorgeloos [32] reported cyst diameter as small as 224 μm for the San Francisco Bay strain (California, USA), while Abatzopoulos *et al*. [33] reported cyst diameters as large as 330 μm for the bisexual species *A. tibetiana*, surpassing even the well known large cyst diameters of the polyploid parthenogenetic strains with a typical diameter near 280 μm. Cysts from Great Salt Lake (GSL-Utah, USA) have a larger cyst diameter (244.2 – 252.5 μm) compared with those from SFB (California, USA) (223.9 – 228.7 μm) [32]; cyst diameters in the Colombian Caribbean are more similar to cysts from GSL than SFB (control). Thus, a cyst diameter grouping in the following order is possible for strains (smallest to largest): 1) San Francisco Bay (SFB-Control ARC1258, USA), 2) Galerazamba and Tayrona, 3) Kangarú, 4) Manaure, and 5) Salina Cero and Pozos Colorados. However, within the same species, strains present different cyst diameter as well as different chorion thickness. The chorion thickness for GSL ranges from 4.7 to 5.7 μm and for SFB it is 7.1 to 8.3 μm [32]. In the case of cyst samples examined in this study, Tayrona were the thinnest, followed by Salina Cero, Galerazamba, Manaure, SFB (USA), Kangarú and Pozos Colorados (see Table 1).

The biometric analysis grouped strains according to naupliar size (Instar I) as follows (smallest to largest): 1) Galerazamba, 2) Manaure, 3) SFB, Kangarú, and Salina Cero, 4) Pozos Colorados, and 5) Tayrona. However, it is remarkable that the naupliar length measured by Vanhaecke and Sorgeloos [32] for the Galerazamba strain collected in 1977 greatly differed from that length in our study (480 ± 31.1 vs. 390.3 ± 24.5 μm); maybe because of some physicochemical effects (i.e. salinity) on the strain manifested over time, and/or food conditions and/or cysts harvested from different pond sizes (widely separated harvest sites), which have been reported [32] to also affect cyst size and chorion thickness. The same authors reported that SFB cysts produced at 180 mg.l^-1 ^*in vitro *are significantly smaller than cysts produced at lower salinities. Biometrical studies performed on several strains from different geographical origins concluded that *Artemia *biometrical parameters were mainly strain specific [32]. These authors revealed that the length of Instar-I nauplii (both bisexual and parthenogenetic) may vary between 430 and 520 μm. Moreover, the North American Instar I nauplii (bisexual) tend to be at the lower previously given range. Thus, naupliar length for SFB (California) is between 428 ± 28.8 to 431 ± 23.7 μm, and for GSL strain (Utah) is between 486 ± 30.6 to 489 ± 29.2 μm [32]. Naupliar size is non-critical for the feeding of crustacean larvae, which can capture and manipulate nutritional particles with their feeding appendages [18]. In contrast, prey size is very critical for fish larvae, which do not have feeding appendages and must engulf particles. The correlation between naupliar size and fish larvae mortality indicates that 20% of the larvae die of hunger when being offered nauplii greater than 480 μm in the first stages of feeding [34]. Thus, depending on the developmental stage of the cultured fish larvae, selecting an appropriate naupliar size as live feed is critical.

Overall, samples from Manaure, Galerazamba and Salina Cero, according to the cyst quality test, exhibited a relatively high number of cysts.g^-1^. H% might have been affected (low in all samples evaluated) because the cyst processing method used (1/3 HP air blower with no heating element or temperature control) could not maintain a constant drying temperature/airflow, or some impurities were still present in samples. H% is dependent on degree of diapause termination, cysts energy content and amount of dead/non-viable/abortic embryos due to improper processing and/or storage [13]. Furthermore, HE reflects three factors: 1) H%, 2) presence of other components (i.e. empty shells, salt, sand, cysts water content), and 3) individual cyst weight. The low hatching synchrony of cysts from Manaure and Salina Cero could be attributed to environmental factors (i.e. raining after cysts were dehydrated, salinity, etc.), and/or as mentioned before improper processing. Significant interactions have been reported among some physicochemical-biotic factors (salinity, percent O_2 _saturation and chlorophyll *a*) and *Artemia *cyst production [35], factors which might affect consequently cyst quality.

The ionic analysis of all locations registered a Cl^- ^anion predominance (Table 3), as expected [36], because of their thalassohaline origin. All hypersaline environments analyzed are suitable habitats for *A. franciscana *development [2]. Colombian hypersaline ecosystems sampled are similar to other American Cl^- ^dominant hypersaline biotopes such as Leslie saltworks (California – USA) [37], La Sal del Rey (Texas – USA) [38] and GSL (Utah – USA) [39].

In nature *Artemia *is found at salinity levels between 60 to 220 g.l^-1 ^(depending on the strain and/or species) and in neutral to alkaline waters, at temperatures generally below 34°C, and at rather low O_2 _levels. The low salinities measured for several months at Pozos Colorados and Salina Cero could hinder overtime *Artemia *production by favoring nauplii production (tolerant to low salinities and pHs) and affecting adults survival [35]. Similarly, the low end pH registered in Galerazamba, Salina Cero and Manaure could affect *Artemia *biomass, and even cyst production overtime. As pH decreases below 7.0 naupliar growth decreases and in adults the overall appearance deteriorates [40]. The same authors concluded that the optimum pH for *Artemia *growth was from 8.0 to 8.5. In the case of cyst, hatching efficiency is greatly compromised at pHs below 8.0 [41]. The low O_2 _levels registered at the Tayrona site could be attributed to the high accumulation of organic matter in the pond from surrounding vegetation. Water temperature was at the upper limit in most sites, with extreme temperatures at Pozos Colorados due to the lack of water circulation and small ponds size (< 0.5 ha). The nutrients (N:P, ideally 15:1) ratio was generally maintained within expected limits, except in Pozos Colorados where it was too high (1:1). The low primary production (chlorophyll *a*) determined in all sites was characteristic of hyperhaline environments. The most photosynthetically productive hypersaline environments [36] are the hypohalines and mesohalines. Furthermore, primary productivity in Salina Cero and Pozos Colorados was the highest among all sites, because of their low salinities. It is widely accepted that salinities higher than 50 g.l^-1 ^hinder considerably primary productivity in hypersaline ecosystems perhaps because of an ionic complex formation of the dissolved macronutrients or because of a generic biologic phenomenon of a drastic specific reduction of microalgae, also occurring at higher salinities [42]. The high precipitation in the southern sites explored (Salina Cero, Galerazamba, Pozos Colorados and Tayrona) affected constantly *Artemia *cyst and biomass production by decreasing salinity and affecting light intensity.

The high EPA and ArA levels (Table 5) for Colombian cysts, determined by EFA analysis, compared to the control (SFB) have great potential for the aquaculture industry. In contrast, the low DHA level content in cysts from the Colombian sites (except Manaure) might be of concern if fed to marine larvae without further DHA enrichment, since DHA deficiency affects neural tissues development [30,31], survival and growth [18], particularly at the larval stage. The very low DHA/EPA ratios (<0.5) for all Colombian sites were as expected with values often lower than 1, but this ratios could be increased up to 7 [15] through the addition of DHA rich emulsions. All strains had a low DHA/ArA ratio, contrasting with a high EPA/ArA ratio (close to dietary ratio 2:1 and 1:1 for marine fish-white bass larvae, respectively [26]). *Artemia *ArA storage/usage mechanism might be similar to that of other marine organisms (marine fishes) which conserve ArA even during periods of starvation [19]. The observed fluctuations in ArA and EPA levels according to FAME analysis between, and even within strains (collected in different months), may be due to year-round variations in the biochemical composition of the primary producers available to adult *Artemia *[15].

## Conclusion

The Colombian *A. franciscana *habitats analyzed are of marine (thalassohaline) origin; thus, all locations were expected to be Cl^- ^rich.

The relatively small nauplii are appropriate for marine larvaeculture. In contrast, the strains from Tayrona, Kangarú, Salina Cero, and Pozos Colorados may be of use but limited to *Artemia *small biomass production quantities, because of the small surface area of their respective locations; *Artemia *could be exploited at these locations for local aquaculture applications.

In general, cyst quality evaluation for Manaure, Salina Cero and Galerazamba strains determined that cysts from these three locations could improve their quality by concentrating efforts on cyst processing techniques. Further, cyst quality might have been affected by interactions among some physicochemical-biotic factors and *Artemia *cyst production in their ecosystem which could be improved by managing some of these key physicochemical-biotic factors and/or infrastructure management (e.g. brine concentration in the different basins and nutrients).

EFA profiles demonstrated that Tayrona, Galerazamba, Manaure and Salina Cero strains are suitable for marine aquaculture because of their high EPA/ArA ratio, but might have to be fortified with DHA rich emulsions depending on the nutritional requirements of the species to be cultured, because of their overall low DHA content.

## Methods

### Study area

**Figure 1:**
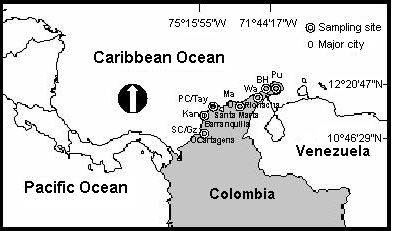
Location of *Artemia franciscana *collection sites: SC: Salina Cero, Gz: Galerazamba, Kan: Kangarú, PC: Pozos Colorados, Tay: Chengue in the Tayrona National Natural Park, Ma: Manaure, Wa: Warrego, BH: Bahía Hondita, and Pu: Pusheo.

*Galerazamba *(10° 47' 38'' N, 75° 14' 48'' W): is a 220 ha thalassohaline saltwork with five ponds, three for brine and two for crystallizers. It is located approximately 20 km North of Cartagena city, at the borderline of the Bolívar department (Fig. 1). Studies have been conducted by several authors in the past using samples from this location [5,11-15,32,35,43-55]. This saltwork, built in a natural saline lagoon and surrounded by mangroves, formed by a sandy-clay and loamy-clay soil type, floods with seawater during high tide throughout the year [45-47].

*Salina Cero or Ciénaga Prieto *(10° 46' 29''N, 75° 15' 55''W): is an 18 ha thalassohaline lagoon 3 km of Galerazamba, Bolivar department (Fig. 1) [11,12,35], studied in September 1998. For many decades, salt has been manually extracted once or twice per year, and fishermen noted the presence of *Artemia *for over five decades.

*Kangarú *(11° 59' 28''N, 74° 32' 21''W): is less than a 4 ha natural thalassohaline saltwork comprised of three small ponds located in the northern region of the Salamanca Island National Natural Park, Magdalena department (Fig. 1). It was explored in July 2000. Salt has been occasionally exploited for decades. This locality, is an important bird migration spot, however, it lost importance because of mangrove destruction as consequence of building a highway through the park.

*Pozos Colorados *(11° 09' 45''N, 74° 13' 34''W): is an approximately 65 ha very old artificial thalassohaline saltwork, currently abandoned. Few studies have been conducted by local researchers in the past using samples from this location. It is located near Santa Marta city, Magdalena department (Fig. 1), contiguous to the road connecting to Barranquilla to Santa Marta city [11]. This saltwork consists of only five irregularly shaped, shallow ponds with only 4 ha water surface.

*Tayrona National Natural Park *(Chengue natural saltwork where the 'Tayrona' *Artemia *population was first reported) (11° 19' 03''N, 74° 08' 13''W): This natural thalassohaline saltwork (Fig. 1) of approximately 2.5 ha is hypersaline due to a closure pattern of dynamic sedimentation of the communication channel with the inlet [54]. It is located in the Magdalena department [11,12,35,53-56]. Tayrona NNP encompasses a small number of saline non crystallizing ponds, with the exception of Chengue, where *Artemia *has been reported to occur. The salt pond is flooded during most of the year and serves as a saltwork during summer [57]. Chengue Inlet, is located in the middle of the Tayrona NNP, it presents a series of small bays and inlets extending from Santa Marta to Cañaverales to the east. Chengue salt exploitation existed long before the prehispanic period [58].

*Manaure *(11° 46' 32''N, 72° 29' 27''W): is located to the west, contiguous to the town of Manaure, in the center of La Guajira department, near Riohacha city (Fig. 1). Studies have been conducted by several authors in the past using samples from this location [5,11-13,35,43,47,48,50,52,54,55,59]. This saltwork is a thalassohaline, shallow water body extending over 4,000 ha. Water movement through the saltwork system is achieved both by pumping and through gravity. There are six pumping stations that increase water volume to a predetermined water level, thereafter water will flow by gravity. This zone was originally a natural lagoon surrounded in some areas by mangroves. The deposits were constructed using the natural topography of the terrain with some modifications. The levees were built by compacting large amounts of clay material brought from the margins of the saltwork [47].

*Warrego *(12° 19'N, 71° 54'W): is an approximately 600 ha (2 miles long) thalassohaline saltern located in the northern tip of La Guajira department, near Puerto Nuevo village (Fig. 1). Occasionally, the Wayu Indians extract salt when the brine crystallizes. Since it was completely dried up when we visited it (January 18, 2000), no water samples were collected from this location and found few *Artemia *cysts.

*Bahía Hondita *(12° 19' 28''N, 71° 44' 13''W): is a natural thalassohaline saltern, approximately 3000 ha, located in La Guajira department (Fig. 1). The Wayu Indians also extract salt in this saltern when the brine crystallizes. We visited the area on January 18, 2000 and only found *Artemia *cysts.

*Pusheo *(12° 20' 47''N, 71° 44' 17''W): is an approximately 400 ha thalassohaline saltern located in the northern tip of La Guajira department (Fig. 1), near Punta Gallinas. Occasionally, the Wayu Indians extract salt when the brine crystallizes. We visited the area on January 18, 2000, and only found *Artemia *cysts.

### Preparation and sampling

Sampling was conducted monthly and cysts batches were collected irregularly (whenever available) in nine thalassohaline locations aforementioned in the northern region of the Colombian Caribbean, from July 1998 to June 2000. Cyst processing was done following these steps: (i) size separation with brine, (ii) density separation in brine, (iii) washing in freshwater, (iv) density separation in freshwater, (v) drying below 40°C, and (vi) vaccum packing and refrigerating cyst at 4 ± 2°C.

Cyst diameter and chorion thickness were recorded from sites where sufficient cysts were collected, using SFB (USA, ARC1258) cysts as reference material. Cysts were incubated for 3 hr in 10 g.l^-1 ^artificial sea water (Instant Ocean^®^) at 25 ± 0.5°C and pH 8.3 [13]. One percent lugol's solution (5 %) was added to the sea water to stop embryos from hatching and cysts were in the dark overnight. *Cyst diameter *(μm) was measured in 200 cysts with a precalibrated microscope. Mean value and standard deviation were calculated using the predetermined conversion factor. *Decapsulated cyst diameter *(μm): a small sample of cysts was hydrated in tapwater for 2 h. Cysts were then decapsulated with a NaOH and NaOCl solution. Cysts were rinsed well and incubated in 10 g.l^-1 ^artificial sea water (Instant Ocean ^®^) with 1 % lugol for 1 hr, at 25 ± 0.5°C, and pH 8.3 and was incubated for 1 h more. Afterwards, 1 % lugol was added again to the incubating solution and cysts were left overnight in the dark.

Decapsulated cyst diameter was measured for 200 cysts with a precalibrated microscope. Mean value and standard deviation were calculated using the predetermined conversion factor. Chorion thickness was calculated using this formula:

(cyst diameter - decapsulated cyst diameter)/ 2

Naupliar length was determined on Instar I nauplii, following this procedure [13]: cysts were incubated and hatched under controlled conditions (25 ± 0.5°C, pH 8.3 and illumination: 1000 lux) in artificial sea water (Instant Ocean^®^) at 35 g.l^-1 ^salinity [32]. Nauplii were sampled at Instar I considering the protopodite of each antennae which bears two endites with a single long bristle attached to each and their brownish-orange color due to yolk presence (Instar II is translucent) as the traits defining this stage [60,61]. Nauplii were harvested when 90% of the total number of hatchable nauplii had been produced [22]. Two hundred nauplii were fixed in lugol's solution (5%) and the length determined using a microscope with a pre-calibrated projection system. Cyst quality studies [13] were performed only on major saltworks. The following parameters were used to evaluate cyst quality:

i) Hatching percentage (H%): number of nauplii that can be produced under standard hatching conditions from 100 full cysts (with embryos).

**H% **= (N × 100)(N+U+E)^-1^

Cysts (1.6 g) were incubated in 800 ml of 32 g.l^-1 ^microfiltered (<1 μm) seawater (Instant Ocean^®^) under continuous illumination (2000 lux) at 28°C, pH = 8.3, in a cylindroconical vessel (test was run in triplicate per strain) with bottom aeration (>2 mg.l^-1^). Vessels were suspended in a water bath in a 100 gal aquarium with a water heather and a mixer to maintain a well distributed temperature (± 1°C). After 24 h incubation six 250 μl subsamples were taken from each cone with a micropipet. Each subsample was pipetted into a small vial and nauplii were fixated by adding a few drops of lugol's solution (5%). Nauplii (n_i_) and umbrella (u_i_) stages were counted in each subsample under a disection microscope. Mean values (N = nauplii and U = umbrella) were calculated each for these two stages. Unhatched cysts were decapsulated and empty cyst shells were dissolved with a drop of NaOH solution (40 g.100 ml^-1 ^distilled water) and five drops NaOCl (5.25% NaOCl) added to each vial. Unhatched (orange colored) embryos (e_i_) were counted per cone (i = 6) and mean value (E) was calculated for each cone. H% value was calculated per cone, and mean value and standard deviation was calculated for three cones (final H% value).

ii) Hatching efficiency (HE): number of nauplii/g dry cysts that can be produced under standard hatching conditions.

**HE **= (N × 4 × 800 ml)(1.6 g)^-1^

HE value was calculated, for each strain evaluated, per cone, and mean value and standard deviation was calculated for three cones (final HE value). Hatching vessels were left for another 24 h, subsequently subsamples were again taken to calculate H% and HE for 48 h incubation period.

iii) Hatching rate (HR): period from incubation (cyst hydration) to nauplii release (hatching). The following HR time intervals are considered:

T_0 _= Incubation time untill appearance of first free swimming nauplii

T_10 _= Incubation time untill appearance of 10 % of total hatchable nauplii

T_90 _= Incubation time untill appearance of 90 % of total hatchable nauplii

T_S _= T_90 _- T_10 _; this value gives an indication of the hatching synchrony

Six 250 μl samples, for each strain evaluated, were taken 12 h after incubation and HE was calculated every 3 h until HE mean value remained constant for three consecutive sampling periods. Mean values per period were then expressed as percentages of the maximal HE. A hatching curve was plotted for each strain, and T_10 _and T_90 _were extrapolated from the graph.

iv) Number of cysts.g^-1^: this parameter is dependent on cysts diameter. Cysts (4 g) were placed in an aluminum plate and weighted and dried in a drying oven set at 60°C for 24 h. Cysts were then cooled down to room temperature for 4 h in a tightly sealed glass drying chamber with fresh desiccant. One g of cyst sample (triplicates) was then weighted (0.1 mg accuracy) in an aluminum plate; to determine average cyst weight, for a single cyst, ten subsamples (10.0 ± 0.1 mg) were taken from each replicate and counted; to find how many cysts were in the 1 g sample average cyst number in 10 mg was then extrapolated to the 1 g cyst sample. This procedure was repeated for each strain evaluated.

At each location we measured: salinity (temperature compensated refractometer), percent O_2 _saturation and temperature (Oxymeter WTW^® ^330), pH (pH meter WTW^® ^330), nitrates, nitrites and phosphates (Hatch^® ^DREL 2010 spectrophotometer), and chlorophyll *a*. For the latter, we used the Seston method and read (Hatch^® ^DREL 2010 spectrophotometer). The ionic composition (Table 3) was determined using a Unicam 939/959 atomic absorption spectrophotometer. All samples were diluted with deionized water because of the high ionic concentration. A sample of nauplii from Galerazamba, Manaure, Salina Cero and Tayrona was taken for FAME; for this analysis we followed Sorgeloos *et al*. [13]: cysts were incubated and hatched under controlled conditions (25 ± 0.5°C, pH 8.3 and illumination: 1000 lux) in artificial sea water (Instant Ocean^®^) at 32 g.l^-1 ^salinity. FAME methodology for freshly hatched nauplii (0.25 g) was a modification of the direct esterification described by Lepage and Roy [62]. The latter implicates a direct acid catalized transesterification without prior extraction of total fat, on dry sample (triplicates) amounts ranging from 10 to 150 mg. An internal standard 20:2 (n-6) was added prior to the reaction. FAME were extracted with hexane. After solvent evaporation FAME were prepared for injection by redissolving them in iso-octane (2 mg/ml). Quantitative determination was done by a Chrompack CP9001 gas chromatograph equipped with an autosampler and a TPOCI (Temperature programmable on-column injector). Injections (0.5 μl) were performed on column into a polar 50 m capillary column, BPX70 (SGE Australia), with a diameter of 0.32 mm and a layer thickness of 0.25 μm, connected to a 2.5 m methyl deactivated precolumn. The carrier gas was H_2_, at a pressure of 100 kPa and the detection mode FID. The oven was programmed to rise from the initial temperature of 85 to 150°C at a rate of 30°C/min, from 150 to 152°C at 0.1°C/min, from 152 to 172°C at 0.65°C/min, from 172 to 187°C at 25°C/min and to stay at 187°C for 7 min. The injector was heated from 85 to 190°C at 5°C/sec and stayed at 190°C for 30 min. Identification was based on standard reference mixtures (Nu-Chek-Prep, Inc., USA). Integration and calculations were done on computer with a software program Maestro (Chrompack).

Any experimental research on animals that was reported in this study was performed with the approval of an appropriate ethics committee regulating animal research.

### Calculations and statistics

Standard deviations were calculated for all cyst diameter and naupliar length measurements. Data obtained were analyzed using one-way ANOVA, and averages compared with Duncan's test (SPSS V10.0).

## Authors' contributions

WNC co-designed and carried out the experiment, participated in data collection, performed data analyses, and wrote manuscript.

GCD, LCH, OCR, JGL and IMM participated in data collection.

OCR evaluated cysts quality.

PS co-designed the experiment, FAME analysis performed at Artemia Reference Center.
